# Tamoxifen induces protection against manganese toxicity by REST upregulation *via* the ER-α/Wnt/β-catenin pathway in neuronal cells

**DOI:** 10.1016/j.jbc.2025.108529

**Published:** 2025-04-23

**Authors:** Alexis Digman, Edward Pajarillo, Sanghoon Kim, Itunu Ajayi, Deok-Soo Son, Michael Aschner, Eunsook Lee

**Affiliations:** 1 Department of Pharmaceutical Sciences, Florida A&M University, Tallahassee, Florida, USA; 2 Department of Biochemistry, Cancer Biology, Neuroscience and Pharmacology, Meharry Medical College, Nashville, Tennessee, USA; 3 Department of Molecular Pharmacology, Albert Einstein College of Medicine, Bronx, New York, USA

**Keywords:** tamoxifen, REST, NRSF, Wnt signaling, β-catenin, manganese, estrogen receptor, neuroprotection

## Abstract

Chronic exposure to elevated levels of manganese (Mn) causes a neurological disorder referred to as manganism, with symptoms resembling Parkinson's disease (PD). The repressor element-1 silencing transcription factor (REST) has been shown to be neuroprotective in several neurological disorders, including PD, suggesting that identifying REST upregulation mechanisms is an important avenue for the development of novel therapeutics. 17β-estradiol (E2) activates the Wnt/β-catenin signaling, which is known to increase REST transcription. E2 and tamoxifen (TX), a selective estrogen receptor modulator, exerted protection against Mn toxicity. In this study, we tested if TX upregulates REST potentially *via* Wnt/β-catenin signaling in Cath.a-differentiated (CAD) neuronal cells using luciferase assay, qPCR, Western blot analysis, immunocytochemistry, mutagenesis, chromatin immunoprecipitation, and electrophoretic mobility shift assay. TX (1 μM) increased REST promoter activities and mRNA/protein levels and attenuated Mn (250 μM)-decreased REST transcription in parallel with TX's protective effects against Mn-induced toxicity, potentially *via* Wnt. TX activated Wnt/β-catenin signaling by preventing β-catenin degradation *via* inactivation of glycogen synthase kinase-3 beta, leading to increased β-catenin levels and its nuclear translocation and binding to T-cell factor/lymphoid enhancer binding factor sites on Wnt-responsive elements (WRE) of the REST promoter. Mutation of WRE abolished TX-induced REST promoter activity. TX-induced Wnt signaling activation was primarily *via* the estrogen receptor (ER)-α, although ER-β and G protein-coupled estrogen receptor 1 also mediated TX's action on REST transcription. These findings underscore the critical role of Wnt/β-catenin signaling in TX-induced REST transcription, affording protection mechanisms against Mn toxicity and neurological disorders associated with REST dysfunction.

Manganism is a neurological disorder caused by chronic exposure to elevated levels of manganese (Mn) *via* occupational settings, such as welding and mining (1), as well as environmental settings, including Mn-contaminated air and drinking water (2, 3, 4). Upon entering the brain, Mn preferentially accumulates in the basal ganglia, such as substantia nigra (SN), globus pallidus (GP), and striatum (5), impairing the dopaminergic (DAergic) neurons in the nigrostriatal pathway, contributing to the dysfunction of motor coordination that were also observed in idiopathic Parkinson's disease (PD) (6). Mn has also been considered one of the environmental risk factors for idiopathic PD (7). Given Mn's ability to disrupt brain function, extensive studies have been conducted to understand the cellular and molecular mechanisms of Mn-induced neurotoxicity. Mn induces mitochondrial dysfunction, oxidative stress, neuroinflammation, apoptosis, and dysregulation of several gene expressions, which are crucial in dopamine (DA) synthesis, including tyrosine hydroxylase (TH) (8, 9, 10). However, precise mechanisms remain incompletely understood.

TH, the rate-limiting enzyme in DA synthesis, is expressed in various brain regions innervating catecholaminergic neurons, such as the striatum, SN pars compacta, ventral tegmental area, hypothalamus, and brainstem (11, 12, 13, 14). Studies have shown that Mn damages DAergic neurons by inducing oxidative stress and apoptosis, as well as dysregulating TH expression and function (15, 16, 17). Mn impairs TH function by activating protein kinase C-δ and increasing protein phosphatase 2A (17), and reduces TH mRNA/protein levels by dysregulating the transcription factor (TF) neuron-restrictive silencing factor/repressor element-1 silencing transcription factor (NRSF/REST), which binds to the RE1 consensus sites in the TH promoter and positively regulates TH (16). In support of the protective role of REST against Mn-induced dysregulation of TH, REST overexpression increased TH mRNA/protein levels and attenuated Mn-decreased TH, oxidative stress, and apoptosis (16). In fact, in addition to Mn, REST induces neuroprotection against several neurodegenerative diseases, such as PD (18) and Alzheimer's disease (AD) (19), while REST deficiency induces genes associated with oxidative stress and apoptosis in PD and AD models (18, 20). Since REST deficiency impairs neuronal functions and is associated with neurodegeneration, investigating the mechanisms of REST upregulation is critical in affording protection against Mn-induced toxicity as well as neurodegenerative diseases associated with REST deficiency.

The activation of the Wnt/β-catenin signaling pathway has been shown to increase REST transcription (21). This signaling activation induces the binding of T-cell factor/lymphoid enhancer binding factor (TCF/LEF) to Wnt-responsive elements (WREs) in the REST promoter, indicating that REST is a direct target of the Wnt/β-catenin pathway (21). Its activation involves the binding of Wnts, such as Wnt3a, to the Frizzled (Fzd) receptor and low-density lipoprotein-related protein 5 and 6 (LRP5/6), leading to the disassembly of the destruction complex, which includes glycogen synthase kinase-3 beta (GSK3β), thereby preventing β-catenin degradation (22). The regulation of GSK3β kinase activity through phosphorylation at different sites of GSK3β controls β-catenin degradation and its nuclear translocation, followed by its binding to the WREs on the target genes (23). The Wnt/β-catenin signaling plays a critical role in central nervous system (CNS) development, regulating neuronal maturation and migration (24, 25, 26, 27). Moreover, Wnt signaling also exerts immunomodulatory functions and anti-inflammatory effects in the brain (28, 29). However, dysregulation of this pathway has been implicated in neurodegenerative disorders, such as AD, PD, cerebral ischemia, Huntington's disease, and multiple sclerosis (for review, (30)), and heavy metal-induced neurotoxicity (31, 32). As a result, research on activating Wnt/β-catenin signaling has emerged as a promising therapeutic strategy against neurodegenerative diseases (for review, (30)). Since Wnt signaling increases REST transcription and Mn reduces it, activation of this Wnt pathway is likely to induce neuroprotection against Mn toxicity. Therefore, identifying pharmacological agents that upregulate the Wnt/β-catenin signaling to enhance REST expression and protect against Mn toxicity is of great interest and highly beneficial in clinical studies.

The female sex hormone 17β-estradiol (E2) is known to exert protective effects in several neurodegenerative diseases (33, 34, 35), as well as manganism (36, 37). E2 induces neuroprotection through both genomic nuclear estrogen receptor (ER) (36) and non-genomic ER signaling, such as the Wnt/β-catenin (38, 39). Since E2 induces peripheral adverse effects, it is imperative to discover or develop E2 agonists that are selectively active in the brain without peripheral effects. Several researchers have coined these compounds or drugs as neuro-selective estrogen receptor modulators (neuroSERMs) (40). To achieve this goal, we investigated tamoxifen (TX), a SERM known for its tissue-specific agonistic and antagonistic effects, to determine if it induces protective effects through the upregulation of REST. TX is widely used to treat breast cancer owing to its antagonistic action in the breast tissues (41, 42, 43). Intriguingly, TX appears to exert neuroprotective effects, as evidenced by a lower risk of dementia and AD in breast cancer patients receiving TX as a treatment (44, 45), as well as PD in both clinical and animal studies (46, 47, 48, 49). In addition, TX exerts neuroprotection in several neurological disorders, including hippocampal silent infarct (SI) in male rats (50, 51), cerebral ischemia in rodent models (52, 53, 54, 55), methamphetamine-induced toxicity in mice (56, 57), and 1-methyl-4-phenyl-1,2,3,6-tetrahydropyridine (MPTP) PD models (58, 59). TX's protective mechanisms include its ability to mitigate SI-induced cognitive dysfunction, apoptosis, gliosis, inflammation, and ER loss in rats (50, 51). Furthermore, TX has been shown to inhibit ischemia-induced phosphorylation of extracellular regulated kinase (ERK) 1/2, oxidative damage, and apoptosis in rat models (52). In methamphetamine-induced toxicity in male mice, TX prevented the loss of DAergic markers and transporters (56, 57). These findings suggest that TX modulates several targets to exert neuroprotection. In fact, TX induces agonistic function in the brain tissues, mimicking E2 effects (49). TX can activate both the genomic and non-genomic pathways to induce its protective effects. TX activates the genomic pathway by binding to cytosolic ER-α and subsequently translocating to the nucleus and binding to the estrogen response elements (ERE) in the promoter region of its target genes (60). On the other hand, its non-genomic effects are mediated by binding to G protein-coupled estrogen receptor 1 (GPER1) (61) and membrane-bound ERs (mER) (54), regulating several signaling pathways, including phosphatidylinositol 3-kinase(PI3K)/Akt/mTOR signaling (62).

Moreover, TX has demonstrated neuroprotective effects against Mn toxicity in both *in vitro* and *in vivo* experimental models (36, 49). Since REST is protective against Mn toxicity (16), in the present study, we investigated if TX increases REST expression levels as a protection mechanism and if TX increases REST *via* activation of Wnt/β-catenin signaling in CAD cells. CAD cells are catecholaminergic neurons that exhibit characteristics of primary neurons (63) by expressing neuron-specific proteins such as class III β-tubulin, GAP-43, SNAP-25, synaptotagmin, and TH. These cells also produce L-DOPA, a precursor of DA, norepinephrine, and epinephrine (63). CAD cells also express L-DOPA decarboxylase (data not shown), suggesting that CAD cells can be used to study DAergic function. SH-SY5Y cells, which are widely used as DAergic cells (64), were used to confirm if the results from CAD cells also occur in DAergic cells by conducting several key experiments. Our findings demonstrate that TX activated the Wnt/β-catenin-REST pathway predominantly *via* ER-α. Moreover, Mn inhibited the Wnt signaling along with REST, while TX attenuated Mn-induced inhibition of Wnt/β-catenin-REST in CAD cells.

## Results

### TX increased REST expression mainly *via* ER-α in CAD cells

**Figure 1:**
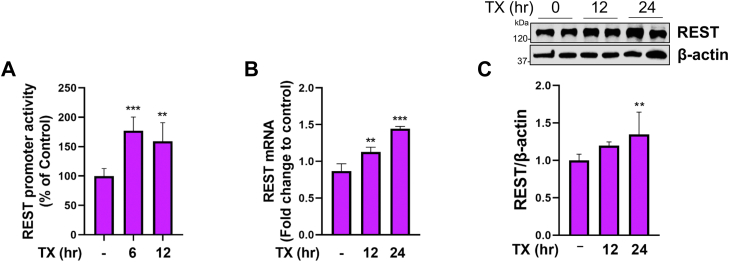
**TX increased REST promoter activity, mRNA, and protein levels in CAD cells.***A*, CAD neurons were transfected with a human 5′UTR-REST promoter vector and then exposed to 1 μM TX, followed by a luciferase assay to measure REST promoter activity. *B* and *C*, CAD cells were treated with 1 μM TX, then followed by measurement of REST mRNA using qPCR (*B*) and REST protein using Western blot (*C*). GAPDH and β-actin were used as loading controls of mRNA and protein, respectively. ∗∗∗*p* < 0.001, ∗∗*p* < 0.01 compared to control. (One-way ANOVA followed by Tukey's *post hoc*, n = 3). The data shown are representative of 3 independent experiments.

**Figure 2:**
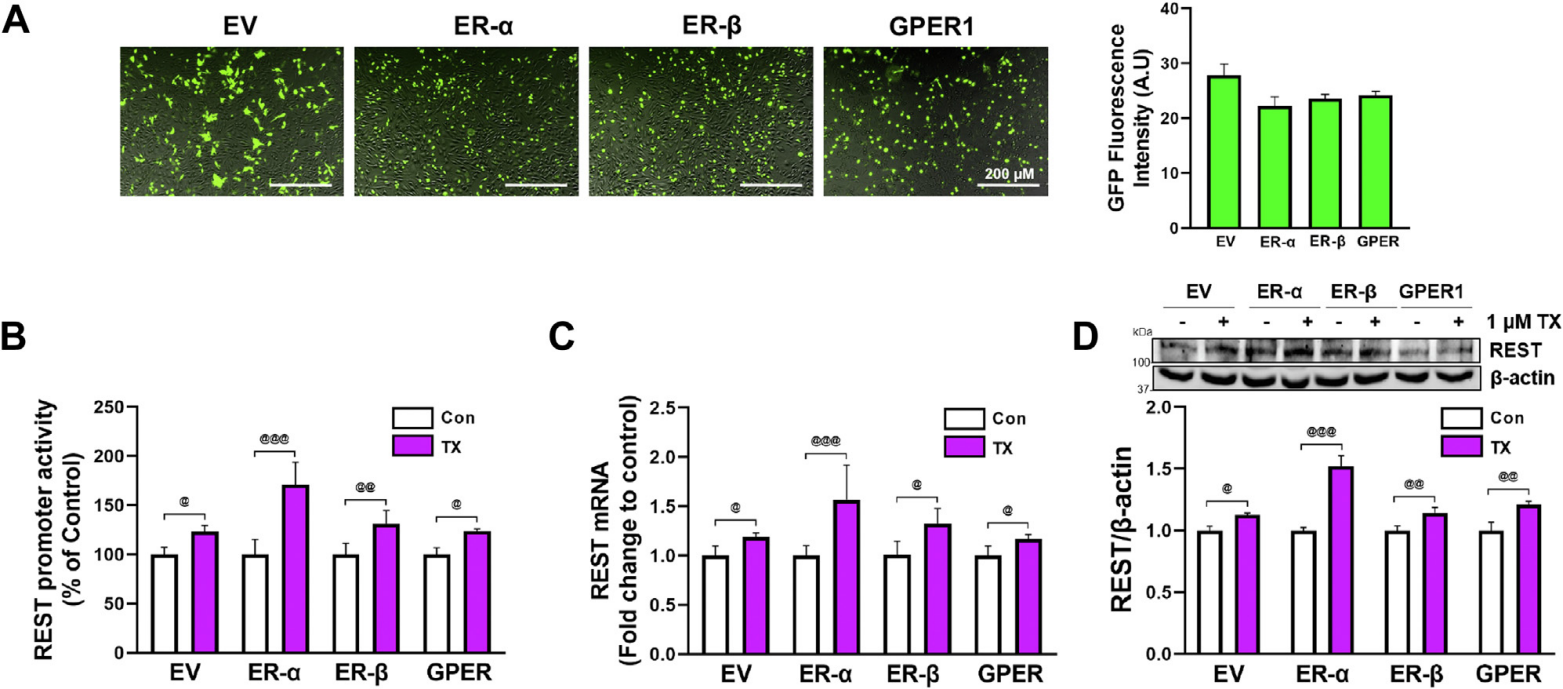
**TX increased REST expression mainly *via* ER-α in CAD cells.***A*, CAD cells were transfected with GFP-tagged empty vector (EV) control, ER-α, ER-β, and GPER1 vectors. Fluorescent images of CAD cells were captured after transfection, and fluorescence intensity was quantified (scale bar, 200 μM). *B*, CAD neurons were co-transfected with human REST promoter and either EV, ER-α, ER-β, or GPER1 expression vectors, then treated with 1 μM TX for 6 h, followed by luciferase assay to detect REST promoter activity. *C* and *D*, Transfected CAD cells were treated with 1 μM TX for 12 h and 24 h to determine REST mRNA (*C*) and REST protein (*D*), respectively. GAPDH and β-actin were used as loading controls of mRNA and protein, respectively. ^@@@^*p* < 0.001, ^@@^*p* < 0.01, ^@^*p* < 0.05 compared to control of each group. (One-way ANOVA followed by Tukey's *post hoc*; n = 3). The data shown are representative of 3 independent experiments.

TX has demonstrated protective effects against neurodegenerative diseases (44, 45, 50) and Mn toxicity (36, 37, 49), but whether TX increases REST transcription *via* Wnt signaling and if activation of Wnt signaling by TX plays a role in REST-mediated protective mechanisms has yet to be established. Therefore, we first determined if TX increased REST expression in catecholaminergic CAD neuronal cells. CAD cells (63) were used for all experiments, and SH-SY5Y cells, which are widely used as DAergic cells (64), were also used to confirm several key findings. The results showed that TX increased REST promoter activity (Fig. 1*A*), mRNA (Fig. 1*B*), and protein levels (Fig. 1*C*) in CAD and SH-SY5Y cells (Fig. S2, *A*–*C*). Given that TX is a SERM, its effects are likely mediated through ERs (65). Thus, we sought to determine which ER subtypes are involved in TX-induced REST upregulation. ER-α, ER-β, and GPER1 were previously identified as ER subtypes (66). To test the involvement of these receptors, CAD cells were transfected with an irrelevant vector control (empty vector or GFP control) or expression vectors of ER-α, ER-β, and GPER1, which were confirmed by the similar protein expression levels of each ER subtype as shown by GFP fluorescence quantification (Fig. 2*A*). The empty vector (GFP control) exhibited higher fluorescence intensity, possibly due to the higher stability of GFP alone over GFP-fusion proteins (67). TX (1 μM, 24 h) was treated in ER-overexpressed CAD cells, followed by an assessment of REST expression. Among the ERs tested, ER-α was found to play the predominant role in mediating TX-induced increase in REST promoter activity (Fig. 2*B*), mRNA (Fig. 2*C*), and protein (Fig. 2*D*) levels in CAD cells.

### TX activated the ER-α/Wnt/β-catenin signaling pathway in CAD cells

**Figure 3:**
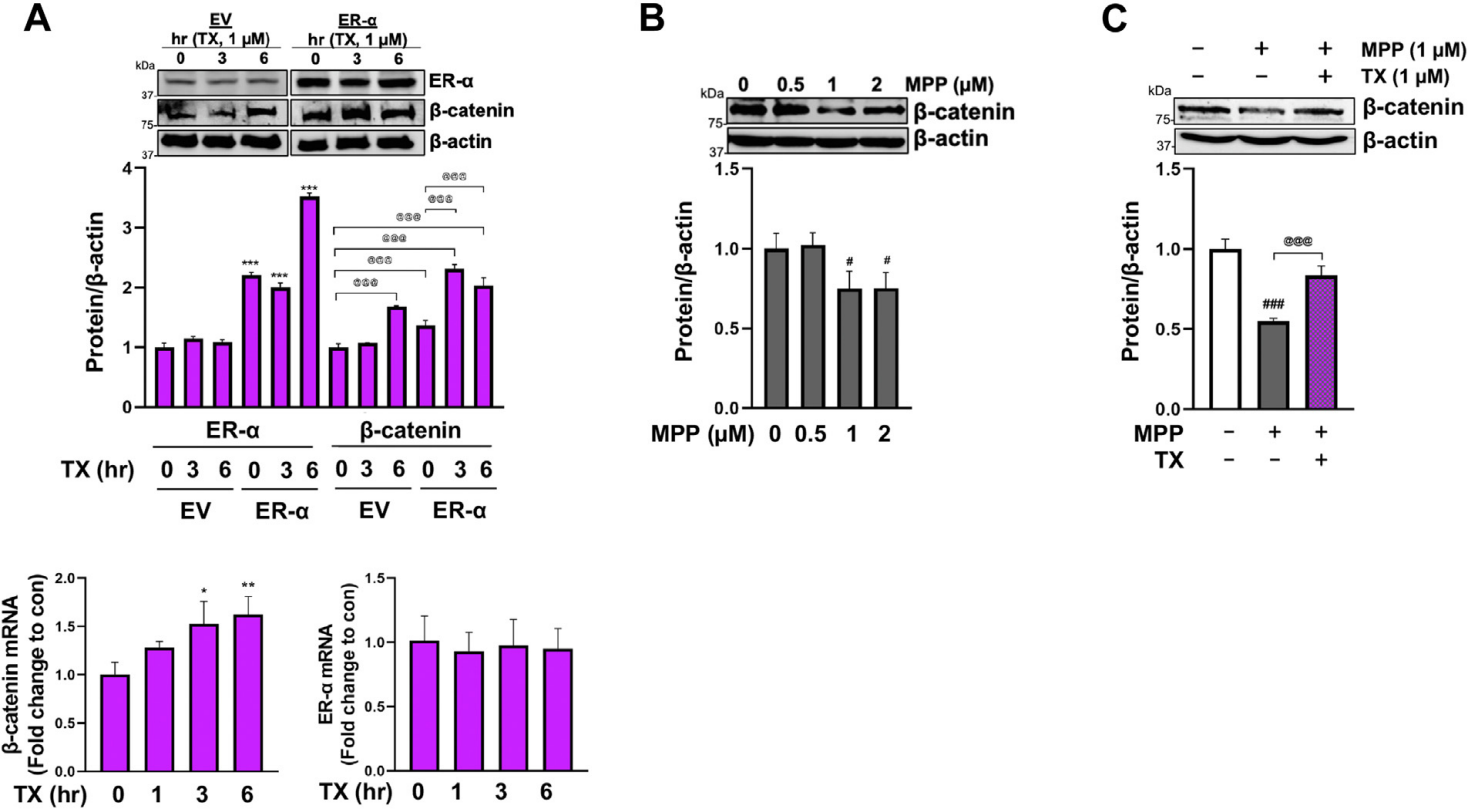
**TX activated the ER-α/Wnt/β-catenin signaling pathway in CAD cells.***A*, CAD were transfected with EV or ER-α vectors, then treated with 1 μM TX, followed by measurement of ER-α and β-catenin protein using Western blot. CAD neurons were treated with 1 μM TX for several time points, followed by ER-α and β-catenin mRNA levels detection using qPCR. *B*, CAD cells were treated with ER-α antagonist MPP for 6 h, followed by measurement of β-catenin protein. *C*, CAD cells were treated with MPP for 6 h prior to 6 h exposure with TX (in the presence of MPP), then subsequently measured β-catenin protein levels. β-actin was used as a loading control of protein. ∗∗∗*p* < 0.001, ∗∗*p* < 0.01, ∗*p* < 0.05, ^###^*p* < 0.001, ^#^*p* < 0.05 compared to control. ^@@@^*p* < 0.001 compared to each other. (One-way ANOVA followed by Tukey's *post hoc*, n = 3). The data shown are representative of 3 independent experiments.

To further investigate the molecular mechanisms underlying the TX-induced REST upregulation, we tested if the non-genomic Wnt/β-catenin signaling pathway is involved potentially through its agonistic action *via* ERs. It is well-established that E2 activates Wnt/β-catenin signaling, promoting neuroprotection in neurons (68, 69) and upregulation of REST (21). Since ER-α was the predominant ER type mediating TX-induced REST expression, we tested if ER-α plays a critical role in TX-induced enhancement of Wnt/β-catenin signaling in CAD neurons. ER-α was overexpressed in CAD cells (Fig. 3*A*), and subsequently, cells were treated with TX, followed by an assessment of β-catenin levels as a key protein of Wnt signaling. The results revealed that TX increased β-catenin mRNA and protein levels with an additional increase in ER-α-overexpressing cells (Fig. 3*A*). Notably, TX did not increase ER-α mRNA levels in CAD cells in 3 h and 6 h of its exposure (Fig. 3*A*). Furthermore, to validate the ER-α-mediated mechanism, the selective ER-α antagonist MPP was used to determine if blocking ER-α would reduce β-catenin signaling. The inhibition of ER-α decreased β-catenin levels (Fig. 3*B*), but did not completely block TX-induced β-catenin increase (Fig. 3*C*), indicating that other ERs, such as ER-β and GPER1, may also mediate TX-induced activation of Wnt/β-catenin signaling.

### TX attenuated Mn-induced cytotoxicity as well as Mn-decreased REST expression in CAD cells

**Figure 4:**
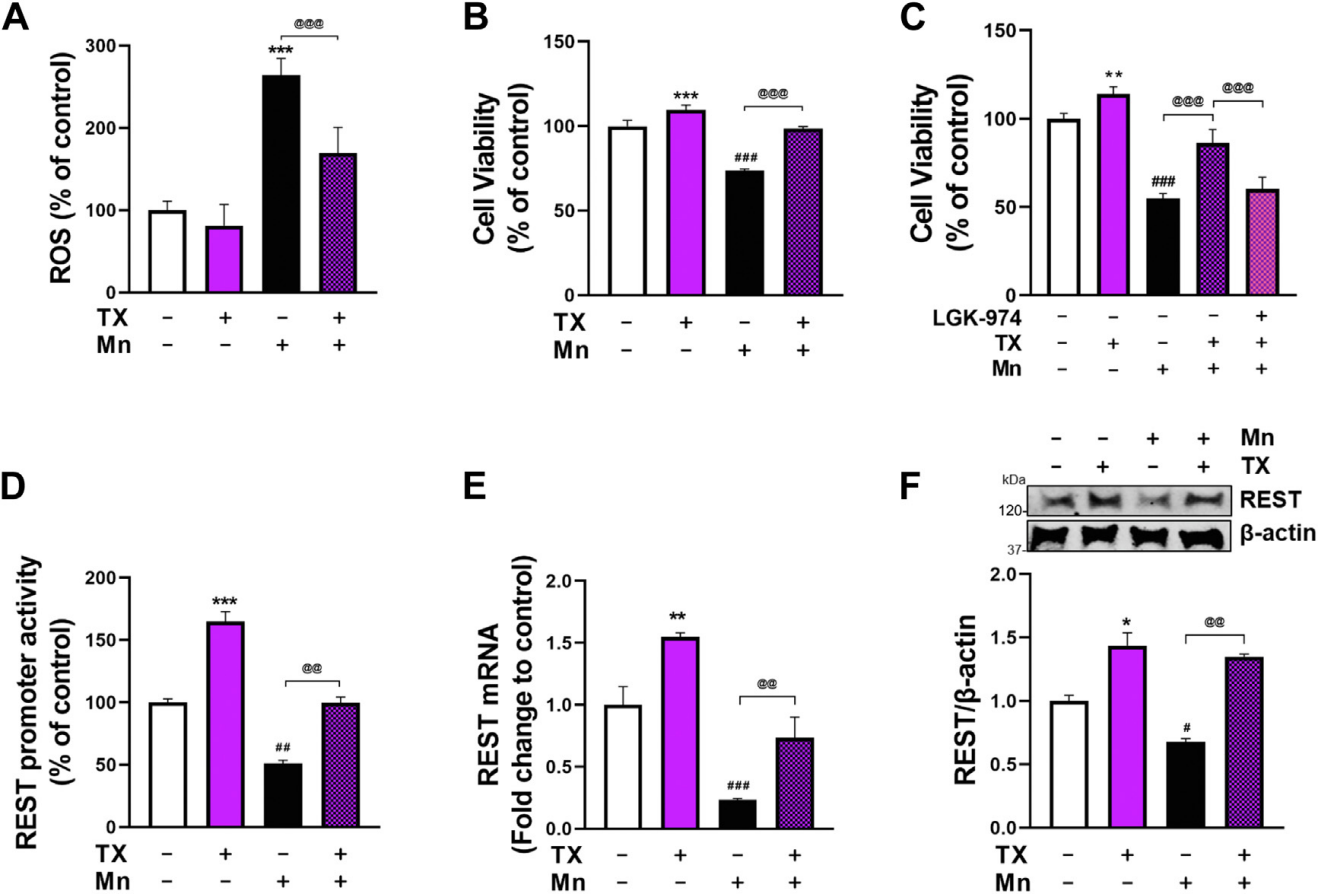
**TX attenuated Mn-induced cytotoxicity as well as Mn-decreased REST expression in CAD cells.***A*, CAD cells were treated with 1 μM TX for 6 h prior to 250 μM Mn exposure for 2 h (in the presence of TX), followed by measurement of ROS levels by a fluorometer using CM-H_2_DCFDA. *B*, CAD cells were treated with 1 μM TX for 24 h, followed by 250 μM Mn exposure for 24 h (in the presence of TX), followed by assessment of cell viability using MTT assay. *C*, CAD cells were treated with 3 μM LGK-974 for 12 h, followed by 1 μM TX for 12 h, then exposed to 250 μM Mn for 24 h (in the presence of LGK-974 and TX). Subsequently, cell viability was assessed using an MTT assay. *D*, CAD cells were transfected with a human 5′UTR-REST promoter vector, then treated with 1 μM TX for 6 h prior to 250 μM Mn exposure for 6 h (in the presence of TX), followed by measurement of REST promoter activity. *E*, CAD were treated with 1 μM TX for 12 h prior to 250 μM Mn exposure for 12 h (in the presence of TX), followed by measurement of REST mRNA. *F*, CAD were treated with TX for 24 h, followed by 250 μM Mn for 24 h (in the presence of TX), followed by measurement of REST protein. GAPDH and β-actin were used as loading controls of mRNA and protein, respectively. ∗∗∗*p* < 0.001, ∗∗*p* < 0.01, ∗*p* < 0.05, ^###^*p* < 0.001, ^##^*p* < 0.01, ^#^*p* < 0.05 compared to control. ^@@@^*p* < 0.001, ^@@^*p* < 0.01 compared to each other. (One-way ANOVA followed by Tukey's *post hoc*; n = 3). The data shown are representative of 3 independent experiments.

Mn induces oxidative stress (16, 70, 71) and decreases cell viability in various cell models (16), and TX possesses antioxidant effects both in neurons and astrocytes (49, 72, 73). The results showed that TX attenuated Mn-induced increase in ROS production (Fig. 4*A*) and also protected CAD cells against Mn-induced cytotoxicity (Fig. 4*B*). In addition, TX's protective effects against Mn toxicity were abolished in the presence of Wnt signaling inhibitor LGK-974 (74) in CAD cells (Fig. 4*C*). These effects were also observed in SH-SY5Y cells (Fig. S2*E*).

Since Mn has been shown to decrease REST expression (16), we tested if TX could mitigate Mn-induced reductions in REST mRNA/protein levels. The results showed that TX attenuated Mn-induced decreases in REST promoter activity (Fig. 4*D*), mRNA (Fig. 4*E*), and protein levels (Fig. 4*F*) in CAD cells, indicating that TX's attenuation of Mn toxicity is in parallel with its restorative effects on REST expression. Although post-treatment with TX after Mn exposure offered some degree of protection, pre-treatment with TX provided greater protection to CAD cells (Fig. S1).

### TX attenuated Mn-induced Wnt3a downregulation and Dkk-1 upregulation

**Figure 5:**
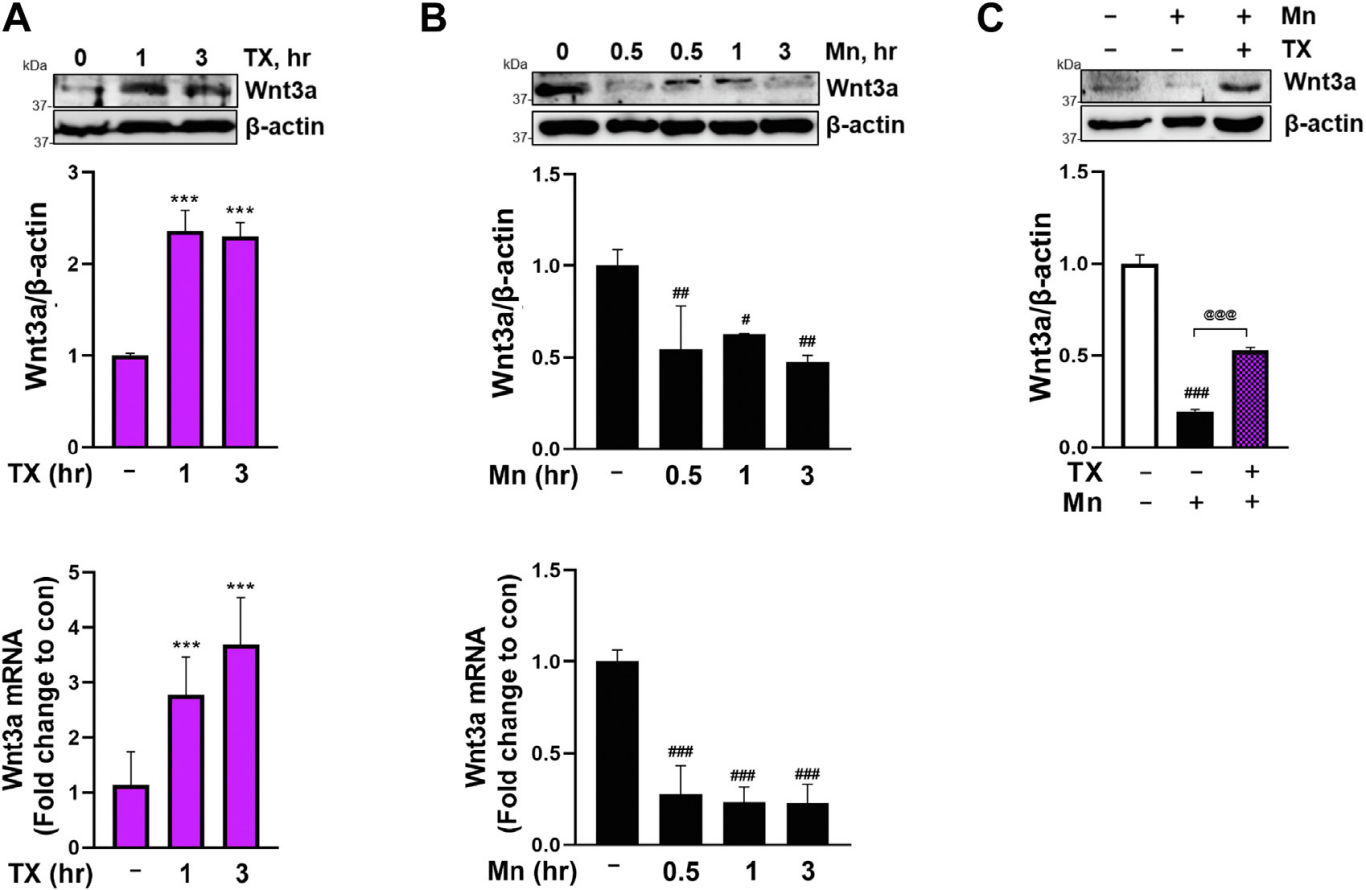
**TX increased Wnt3a and attenuated Mn-induced Wnt3a downregulation in CAD cells.***A* and *B*, CAD cells were treated with 1 μM TX (*A*), and 250 μM Mn (*B*), followed by measurement of Wnt3a protein and mRNA using Western blot and qPCR, respectively. *C*, CAD were treated with 1 μM TX for 1 h prior to 250 μM Mn for 3 h (in the presence of TX), followed by measurement of Wnt3a protein. β-actin and GAPDH were used as loading controls for protein and mRNA, respectively. ∗∗∗*p* < 0.001, ^###^*p* < 0.001, ^##^*p* < 0.01, ^#^*p* < 0.05 compared to control. ^@@@^*p* < 0.001 compared to the Mn-treated group. (One-way ANOVA followed by Tukey's *post hoc*; n = 3). The data shown are representative of 3 independent experiments.

Since REST is a direct target gene of the Wnt/β-catenin signaling pathway (21), we further explored the molecular mechanisms of TX-induced REST transcription. We have tested if TX activates the Wnt/β-catenin pathway through its ER agonistic action, similar to E2, to upregulate REST, by testing several upstream signaling proteins, such as Wnt3a and Dkk-1, which regulate Wnt/β-catenin signaling (75, 76, 77, 78). Wnt3a, a member of the Wnt1 protein family, is known for mitigating toxicity in various cellular and mouse models (22, 75). Notably, E2 has been shown to be neuroprotective against global cerebral ischemia (GCI) by increasing Wnt3 expression in CA1 hippocampal neurons (79). However, whether TX modulates Wnt3 has not been studied. The results showed that TX increased Wnt3a mRNA and protein levels and attenuated Mn-decreased Wnt3a in CAD cells (Fig. 5, *A*–*C*).

**Figure 6:**
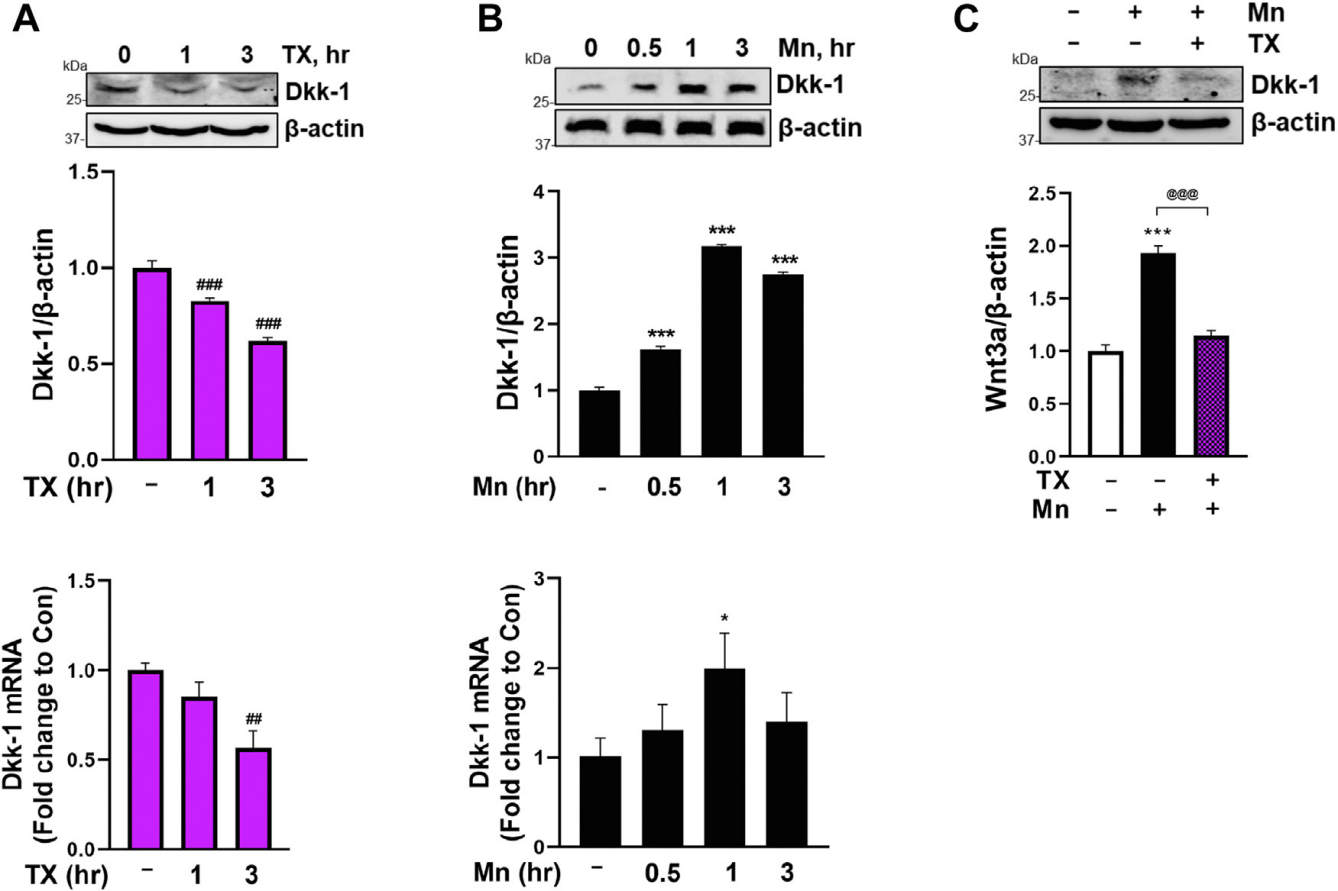
**TX decreased Dkk-1 and attenuated Mn-induced Dkk-1 upregulation in CAD.***A* and *B*, CAD cells were treated with 1 μM TX (*A*), and 250 μM Mn (*B*), followed by measurement of Dkk-1 protein and mRNA using Western blot and qPCR, respectively. *C*, CAD cells were treated with 1 μM TX for 1 h prior to 250 μM Mn for 3 h (in the presence of TX), followed by measurement of Dkk-1 protein. β-actin and GAPDH were used as loading controls for protein and mRNA, respectively. ∗∗∗*p* < 0.001, ∗*p* < 0.05, ^###^*p* < 0.001, ^##^*p* < 0.01 compared to control. ^@@@^*p* < 0.001 compared to the Mn-treated group. (One-way ANOVA followed by Tukey's *post hoc*, n = 3). The data shown are representative of 3 independent experiments.

Dyresgulation of Dkk-1, a secreted inhibitor of β-catenin-dependent Wnt signaling (80), is closely associated with several neurodegenerative disorders (81). E2 has also been shown to attenuate ischemia-induced Dkk-1 elevation (79). Our findings revealed that Mn increased Dkk-1 mRNA and protein levels (Fig. 6*B*), leading to inhibition of Wnt signaling, while TX decreased Dkk-1 mRNA and protein and attenuated Mn-increased Dkk-1 protein levels (Fig. 6, *A* and *C*), restoring the activation of Wnt signaling.

### TX attenuated Mn-induced β-catenin degradation by modulating GSK3β activity

**Figure 7:**
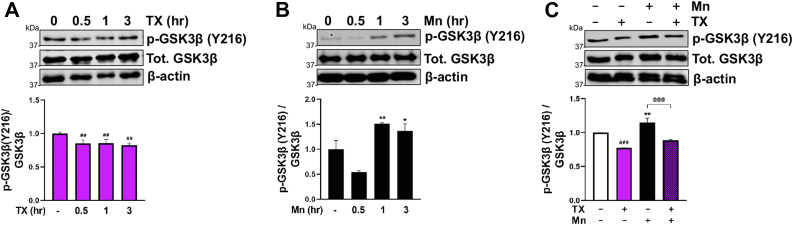
**TX blocked Mn-induced activation of GSK3β by modulating phosporylation at the Y216 site.***A* and *B*, CAD cells were treated with 1 μM TX (*A*) and 250 μM Mn (*B*), followed by measurement of phosphorylation of Y216 on GSK3β (p-GSK3β Y216) using Western blot. *C*, CAD were treated with 1 μM TX for 1 h prior to 250 μM Mn exposure for 1 h (in the presence of TX), followed by measurement of p-GSK3β Y216 protein levels using Western blot. β-actin was used as a loading control of protein. ∗∗*p* < 0.01, ∗*p* < 0.05, ^###^*p* < 0.001, ^##^*p* < 0.01 compared to control. ^@@@^*p* < 0.001 compared to Mn-treated group. (One-way ANOVA followed by Tukey's *post hoc*, n = 3). The data shown are representative of 3 independent experiments.

**Figure 8:**
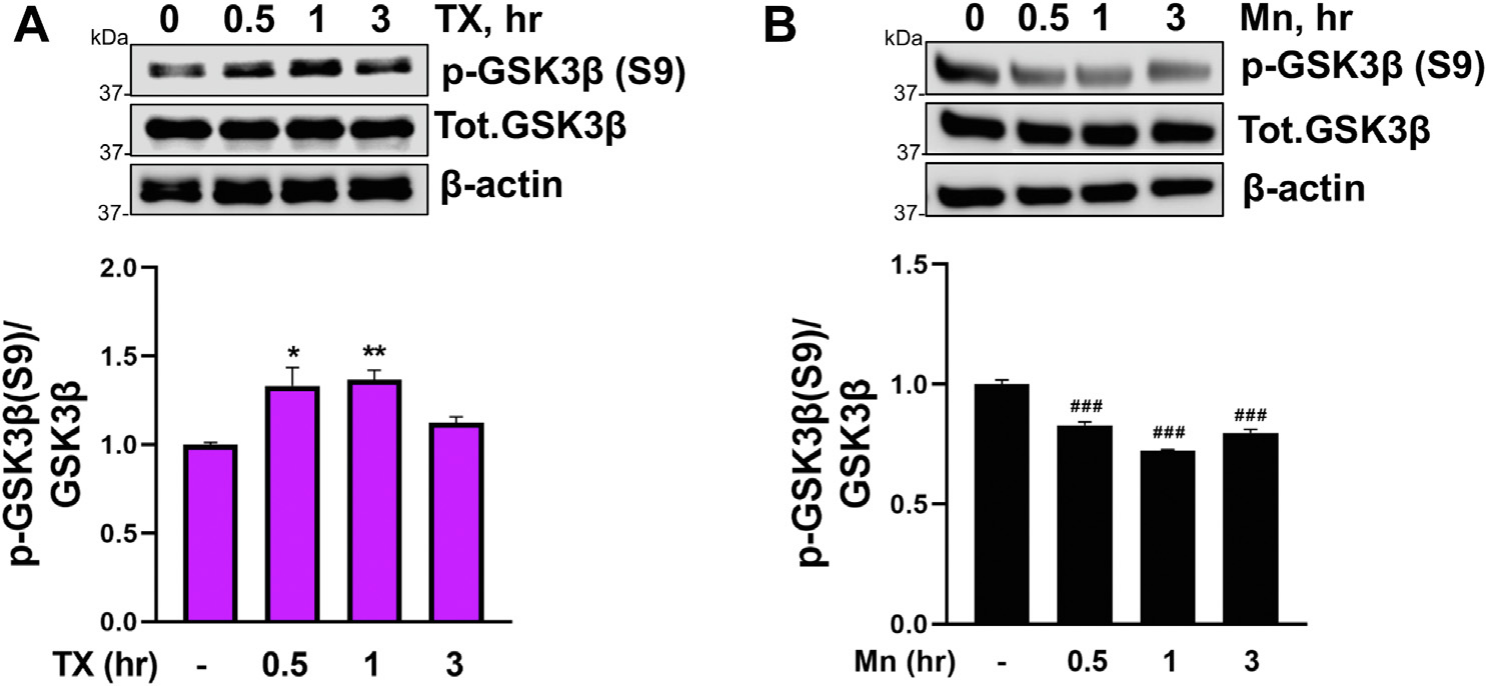
**TX increased, while Mn decreased, inactive GSK3β by modulating phosphorylation at the S9 site.***A* and *B*, CAD cells were treated with 1 μM TX (*A*), and 250 μM Mn (*B*), followed by measurement of phosphorylation of S9 on GSK3β (p-GSK3β S9). β-actin was used as a loading control of protein. ∗∗*p* < 0.01, ∗*p* < 0.05, ^###^*p* < 0.001 compared to control. (One-way ANOVA followed by Tukey's *post hoc*, n = 3). The data shown are representative of 3 independent experiments.

Activation of GSK3β, a component of a protein destruction complex, plays a key role in regulating β-catenin degradation by phosphorylation of GSK3β at the Y216 site (p-Y216), promoting β-catenin phosphorylation and subsequent degradation. In contrast, phosphorylation of GSK3β at S9 inhibits β-catenin degradation (82, 83, 84). In the present study, we found that Mn phosphorylated Y216 (Fig. 7*B*), while TX alone decreased p-Y216 of GSK3β (Fig. 7*A*) and attenuated the Mn-increased p-Y216 of GSK3β (Fig. 7*C*). In addition, Mn also decreased p-S9 of GSK3β (Fig. 8*B*), resulting in β-catenin degradation, while TX increased p-S9 (Fig. 8*A*), leading to inhibition of β-catenin degradation.

**Figure 9:**
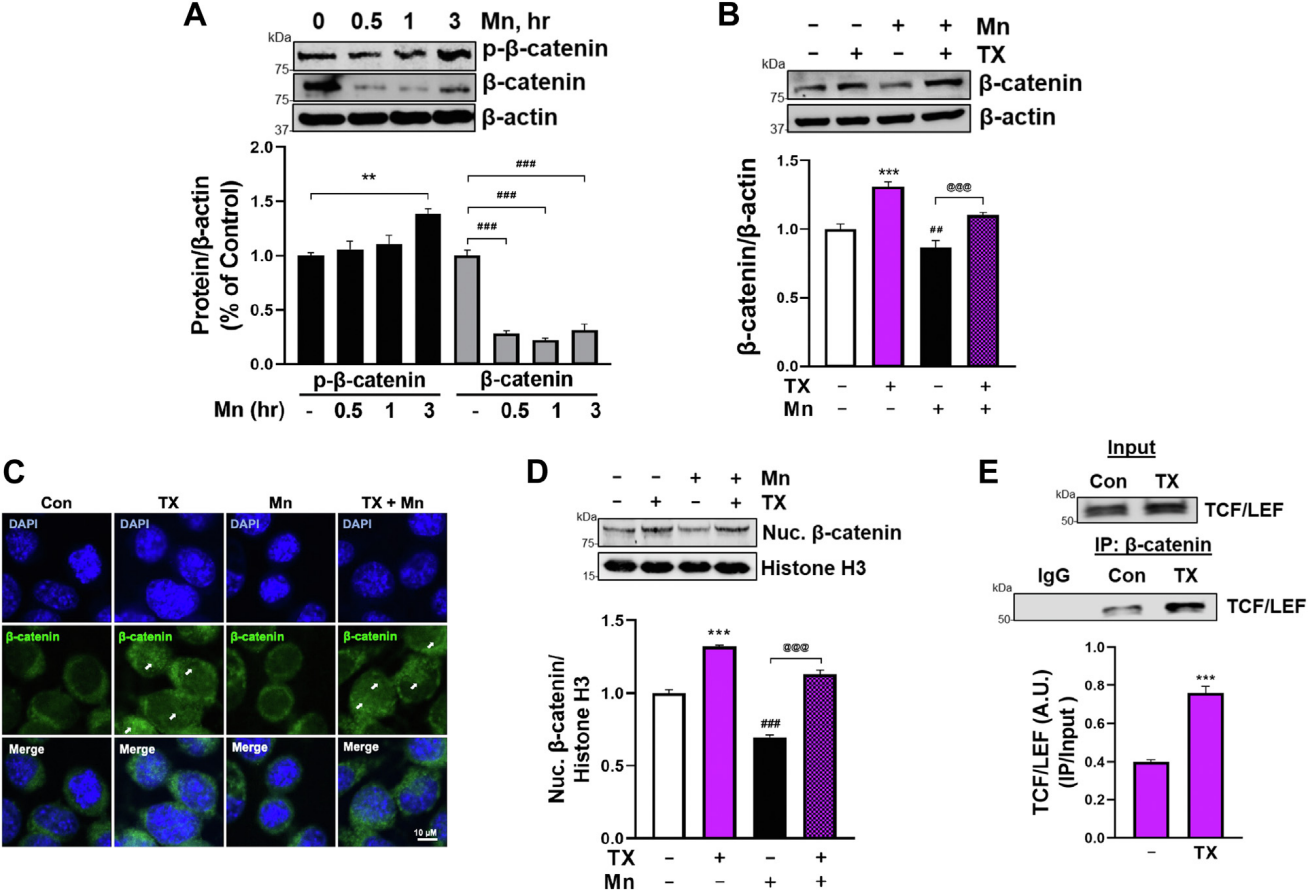
**TX increased nuclear translocation of β-catenin and its interaction with TCF/LEF.***A*, CAD cells were exposed to 250 μM Mn, followed by measurement of phosphorylation of β-catenin on S33, S37, and T41 residues, along with the total β-catenin protein levels using Western blot. *B*, CAD cells were treated with 1 μM TX for 1 h prior to 250 μM Mn exposure for 6 h (in the presence of TX), followed by measurement of total β-catenin protein. *C*, immunofluorescence images of β-catenin nuclear translocation upon incubation of 1 μM TX for 3 h prior to 250 μM Mn treatment for 6 h (in the presence of TX) in CAD neurons (scale bar, 10 μM). *D*, Nuclear β-catenin protein levels were measured from nuclear extracts from CAD cells after treatment of 1 μM TX for 3 h, followed by Mn exposure for 6 h (in the presence of TX) using Western blot. *E*, Nuclear extracts from TX-treated CAD neurons were pulled down using β-catenin antibody, followed by quantification and assessment of its interaction with WRE-bound TCF/LEF TF. β-actin was used as a loading control of total protein extract and histone H3 for nuclear fraction. ∗∗∗*p* < 0.001, ^###^*p* < 0.001, ^##^*p* < 0.01, compared to control. ^@@@^*p* < 0.001 compared to Mn-treated group. (Student's *t* test or one-way ANOVA followed by Tukey's *post hoc*, n = 3). The data shown are representative of 3 independent experiments.

We next investigated if Mn induced β-catenin degradation. The results showed that Mn increased GSK3β-induced phosphorylation of β-catenin, leading to decreased β-catenin protein levels (Fig. 9*A*). Importantly, TX attenuated Mn-induced β-catenin degradation (Fig. 9*B*) by GSK3β modulation in CAD cells. TX's attenuation on Mn-induced β-catenin degradation was also observed in SH-SY5Y cells (Fig. S2*D*).

### TX increased nuclear translocation of β-catenin and its interaction with TCF/LEF

TX-activated Wnt/β-catenin signaling resulted in an increase of cytosolic β-catenin protein levels, and β-catenin subsequently translocated to the nucleus, forming a complex with TCF/LEF. This complex then binds to the WRE of the target genes and modulates transcription of the Wnt target genes (40). Our results showed that TX increased the nuclear translocation of β-catenin and attenuated Mn-decreased β-catenin translocation (Fig. 9, *C* and *D*). Moreover, TX increased the formation of the β-catenin/TCF/LEF complex, which binds to WRE on the REST promoter (Fig. 9*E*).

### TX attenuated Mn-induced decrease of β-catenin binding to the WRE of the REST promoter

**Figure 10:**
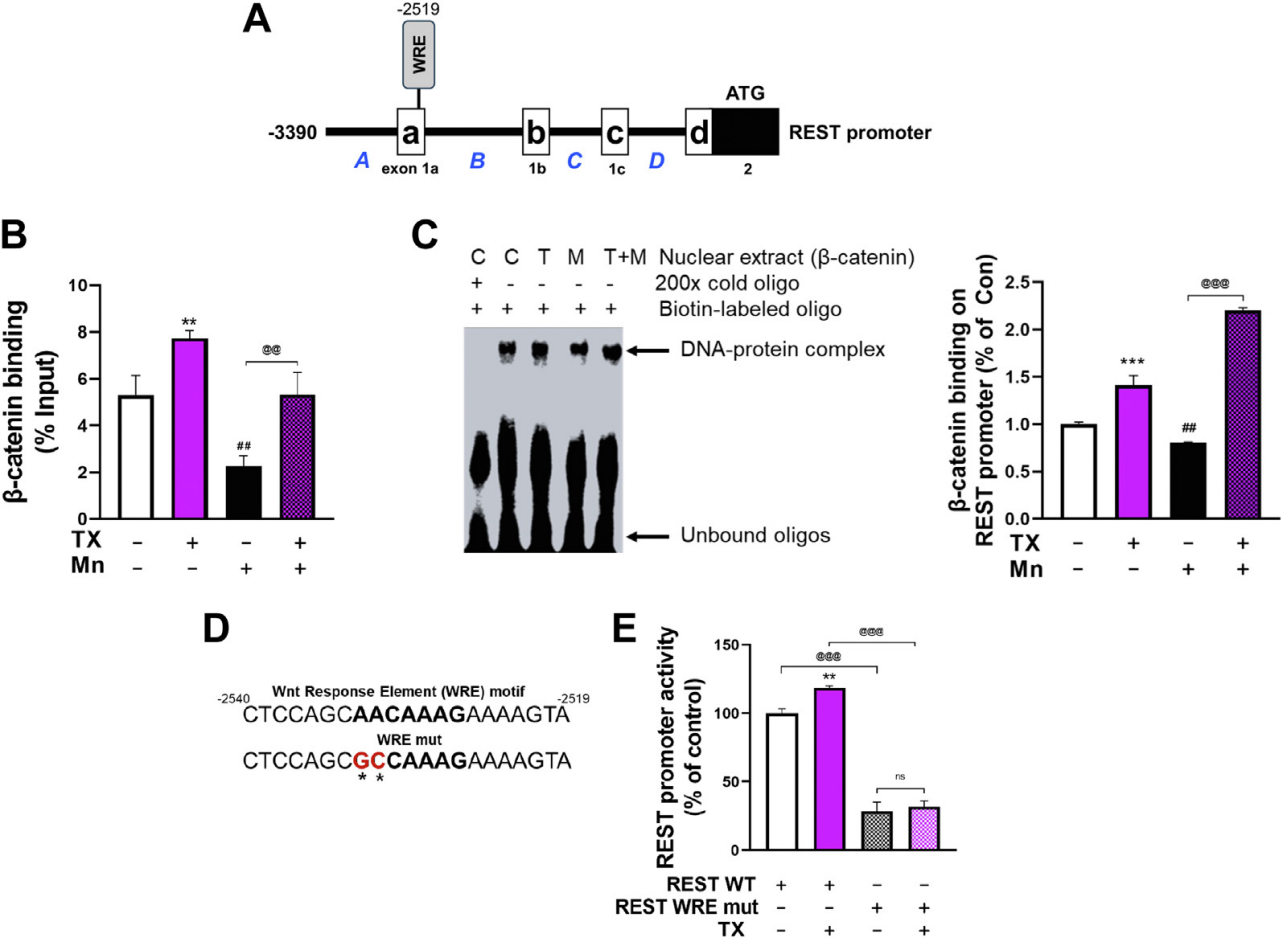
**TX attenuated Mn-induced decrease of β-catenin binding to the WRE of the REST promoter.***A*, illustration of the human 5′ UTR (−3390/+1) REST promoter region with WREs. *B*, after CAD cells were treated with 1 μM TX for 12 h, followed by 250 μM Mn exposure for 24 h (in the presence of TX), ChIP assay was performed to determine the binding of β-catenin on the WREs on the REST promoter *in vivo*, followed by quantification of β-catenin-bound DNA by real-time qPCR. *C*, EMSA was performed in nuclear extracts prepared from CAD cells treated with 1 μM TX for 12 h, followed by 250 μM Mn exposure for 24 h (in the presence of TX) as described in the Experimental procedures. The *black arrow* shows the DNA–protein complex, which was quantified using ImageLab Software (BioRad). *D*, two site mutations of the WRE consensus sequences are indicated in *red*. *E*, CAD neurons were transfected overnight with wild-type human 5′ UTR REST promoter (REST WT) or WRE mutants of REST promoter plasmid (REST WRE mut), then subsequently treated with 1 μM TX for 6 h, followed by luciferase assay. ∗∗∗*p* < 0.001, ∗∗*p* < 0.01, ^##^*p* < 0.01, compared to control. ^@@@^*p* < 0.001, ^@@^*p* < 0.001 compared to each other. (One-way ANOVA followed by Tukey's *post hoc*, n = 3). The data shown are representative of 3 independent experiments.

The human and mouse REST promoters contain the WRE (AACAAAG) sequences in the exon 1A region (−2519), where the β-catenin/TCF/LEF complex binds to regulate its transcription (Fig. 10*A*) (21). To determine if TX or Mn modulates the binding of β-catenin/TCF/LEF to the WRE, ChIP and EMSA were performed. The results revealed that TX increased the binding of the β-catenin/TCF/LEF complex to the WRE of the REST promoter and mitigated Mn-induced decreases of this binding (Fig. 10, *B* and *C*). We also mutated the WRE sequences to further confirm if the WRE is critical for TX effects on REST transcription. The WRE sequences of AACAAAG were mutated to GCCAAAG in the human REST promoter plasmid (Fig. 10*D*) (85). The results showed that the mutation on the WRE sequences markedly reduced REST promoter activity compared to the non-mutated WRE control and also abolished TX-increased REST promoter activity (Fig. 10*E*).

## Discussion

The findings of the present study reveal, for the first time, that TX activates the Wnt/β-catenin signaling pathway, leading to REST upregulation in CAD cells, and attenuating Mn-induced inhibition of this pathway. Activation of the Wnt/β-catenin-REST pathway contributed to the TX-induced protective effects against Mn toxicity, as inhibition of Wnt signaling abolished TX's protection against Mn-induced cytotoxicity. Among the ER subtypes, ER-α emerged as the primary mediator of TX-induced activation of the Wnt/β-catenin-REST pathway, corroborating previous studies where ER-α played a critical role in TX-induced protection against ischemic and traumatic brain injury mouse models (54, 86, 87). Moreover, the mutation of the WRE in the REST promoter abolished TX-induced REST transcription, further confirming that the Wnt/β-catenin pathway is critical in TX-induced REST expression and TX's potential protection mechanism against Mn toxicity.

TX upregulated REST and attenuated Mn-induced REST reduction in CAD cells, suggesting that TX's protective mechanism against Mn toxicity involves REST upregulation. The role of REST protection is in line with the previous study in that REST increases gene expressions associated with oxidative stress resistance and antioxidant enzymes, such as catalase and SOD1 (19). Moreover, the results that Wnt signaling plays a critical role in the upregulation of REST and TX-induced protection against Mn toxicity are corroborating with the previous study showing that inhibition of Wnt signaling increased apoptosis in *in vitro* neuronal cultures (88).

Our findings suggest that TX exerts the ER agonistic effects by activating Wnt/β-catenin signaling *via* ER-α in CAD cells (Fig. 3), as E2 activates Wnt/β-catenin signaling in neurons (68, 69, 79). The critical role of ER-α in TX's effects is also in line with other studies showing that ER-α mediates TX's protective effects in ischemic models of PC12 cells and rats (54), traumatic brain injury model of rats (87), and TX-induced bone regulations in mice (86). Although ER-α is the main ER subtype for TX-induced Wnt/β-catenin signaling, our results suggest that other ER subtypes, such as ER-β and GPER1, also contribute to TX-induced increased β-catenin protein levels. In addition to TX, another SERM, raloxifene, also activated several ER subtypes in increasing glutamate transporter 1 (GLT-1) expression in astrocytes (89), suggesting that SERMs may activate multiple ER subtypes. However, the specific ER subtypes involved in SERMs' effects may vary depending on the cellular context and microenvironment.

The nuclear ERs primarily modulate the transcription of its target genes, but the nongenomic ER signaling *via* the mERs can also regulate transcription ((90) for review). In the brain, the nongenomic ER signaling is activated by GPER1 and mERs such as ER-α and ER-β ((66) for review). These mERs can activate multiple intracellular signaling, including protein kinase A (PKA) and PKC pathways, as well as insulin-like growth factor-1 and Wnt/β-catenin signaling (91, 92, 93, 94, 95, 96). Particularly, mER-α has been shown to activate Wnt signaling to induce osteoblast differentiation in bone (97). Therefore, our findings suggest that TX activates Wnt signaling through mER-α consistent with previous observations that synthetic estrogen activated Wnt signaling *via* mER-α (97).

The upstream mechanism by which TX activates and counteracts Mn-induced inhibition of Wnt/β-catenin signaling is not well understood. It may involve modulation of Wnt3a and Dkk-1 as shown by the results that TX increased Wnt3a and attenuated Mn-decreased Wnt3a in CAD cells (Fig. 5), corroborating with the previous study that Wnt3a exerts protection against 6-hydroxydopamine (6-OHDA) toxicity in SH-SY5Y neurons (75). Mn-increased Dkk-1, an inhibitor of Wnt signaling (98), may contribute to its toxic effects, supported by the previous findings that increased Dkk-1 was involved in the 6-OHDA-induced neurodegeneration in rats (99), as well as in MPP^+^-induced toxicity in PC12 cells (77). The results that TX decreased and attenuated Mn-induced Dkk-1 (Fig. 6) suggest that TX also regulates Dkk-1 in addition to Wnt3a, which may afford a protective mechanism against Mn-induced toxicity. Our findings also showed that TX/Mn modulated both mRNA and protein levels of Wnt3a and Dkk-1, suggesting that these proteins were altered at the transcriptional level.

GSK3β regulates β-catenin degradation by phosphorylation of GSK3β at Y216 which leads to β-catenin degradation, whereas phosphorylation of GSK3β at S9 inhibits its degradation. The results showed that TX and Mn modulated GSK3β activation by regulating phosphorylation at both Y216 and S9 sites (Figs. 7 and 8). Mn decreased p-S9 and increased p-Y216, in line with previous studies that Mn decreased p-S9 levels, resulting in β-catenin degradation in rat striatum and PC12 cells (32). A PD toxin rotenone also modulated both Y216 and S9 phosphorylation sites, leading to β-catenin degradation and cell death in SK-N-MC neuroblastoma cells (100). On the other hand, TX also modulated GSK3β by exerting the opposite effects by decreasing p-Y216 and increasing p-S9, thus counteracting the Mn-induced increase in β-catenin degradation in CAD cells. TX's modulatory effects on GSK3β is likely *via* the ER activation as E2 regulated both S9 and Y216 sites in inducing protection in AD models (96). Our findings reveal that TX not only inhibits Mn-induced β-catenin degradation but also increases β-catenin mRNA levels in CAD cells which corroborates the previous study showing that E2 upregulates β-catenin mRNA in human uterine cells (101).

TX-increased cytosolic β-catenin translocates to the nucleus, and forms β-catenin/TCF/LEF complex, resulting in binding to WREs on the REST promoter to increase REST transcription. The REST promoter contains WRE binding motif (AACAAAG) on exon 1A which is critical for REST expression (21). TX-induced REST transcription appears to be mediated by this WRE site since TX increased β-catenin binding to the WRE of the REST promoter, while mutations at this WRE site abolished TX-induced REST transcription.

**Figure 11:**
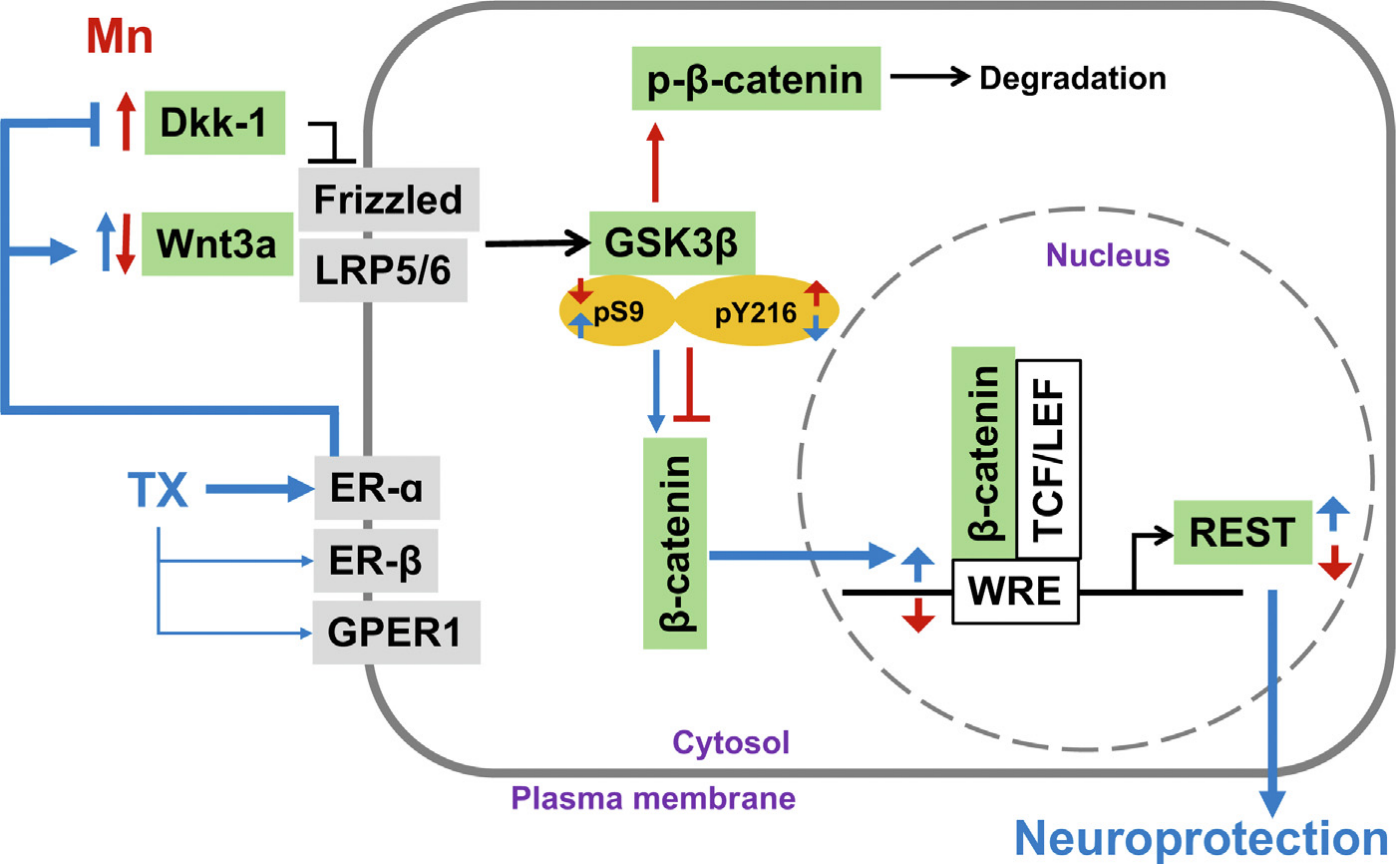
**Schematic diagram of the proposed mechanism of TX**'**s neuroprotection against Mn toxicity by upregulating REST expression *via* Wnt/β-catenin signaling in CAD neurons.** TX activates the Wnt/β-catenin signaling pathway *via* ER-α, while Mn inhibits this pathway by dysregulating Wnt3a and Dkk-1. TX and Mn modulate GSK3β function by differentially phosphorylating GSK3β at S9 and/or Y216, leading to alteration of β-catenin levels in the cytosol and subsequent translocation to the nucleus. β-catenin forms a complex with TCF/LEF in the nucleus, which binds to WRE on the REST promoter, inducing REST transcription. These findings suggest that TX exerts neuroprotective effects against Mn toxicity by increasing REST *via* the ER-α/Wnt/β-catenin signaling pathway. *Red*: Mn-induced effects; *blue*: TX-induced effects.

In summary, our novel findings demonstrate that TX activates the Wnt/β-catenin pathway *via* ER-α, while Mn inhibits this pathway by dysregulating Wnt3a and Dkk-1. Both TX and Mn modulated GSK3β activity by differential regulation of GSK3β phosphorylation. Moreover, TX increased REST transcription and attenuated Mn-reduced REST by activation of the ER-α/Wnt/β-catenin signaling pathway in CAD cells (Fig. 11). Given that REST upregulation is known to induce neuroprotection in various neurological disorders, including Mn-induced toxicity and neurodegenerative diseases, the development of neuroSERMs targeting REST upregulation in a brain neural cell-specific manner absent of peripheral effects might offer a promising approach for novel therapeutic intervention that mitigate these neurological disorders.

## Experimental procedures

### Materials

Manganese (II) chloride (MnCl_2_), TX, retinoic acid (RA), REST antibody (07-579), and dimethyl sulfoxide (DMSO) were purchased from MilliporeSigma. LGK-974 was obtained from Selleckchem Chemicals (Houston, TX). Methyl-piperidino-pyrazole (MPP) dihydrochloride was purchased from Tocris Bioscience (Minneapolis, Mn). All cell culture media, including trypsin-EDTA, minimum essential media, and Dulbecco's modified Eagle's medium (DMEM/F-12) were obtained from Gibco (Thermo Fisher Scientific). Antibodies for phospho-β-catenin on serine 33 and 37, and threonine 41 (9561T) were from Cell Signaling Technology. Antibodies for β-catenin (sc-59737), phospho-GSK3β tyrosine 216 (sc-81496), phospho-GSK3β serine 9 (sc-373800), GSK3β (sc-377213), Dickkopf-1 (Dkk-1, sc-374574), Wnt3a (sc-136163), ER-α (sc-543), and β-actin (sc-47778) were obtained from Santa Cruz Biotechnology. Antibodies for horseradish peroxidase (HRP)-conjugated rabbit anti-mouse IgG (ab-6728), HRP-conjugated goat anti-rabbit IgG (ab-6721), and goat anti-mouse antibodies conjugated with Alexa Fluor 488 (ab-150113) were from Abcam. All chemicals were prepared in phosphate-buffered saline (PBS), double-distilled H_2_O, or DMSO, and diluted to working concentrations in Minimum essential medium (MEM) prior to use. Expression vectors for β-catenin and empty control vector pCDNA3.1 were from Addgene. The human REST promoter plasmid vector was a gift from Dr Yvon Trottier.

### Cell culture

The murine catecholaminergic CAD neuronal cell line (ECACC 08100805) was obtained from MilliporeSigma. CAD cultures were maintained in DMEM/F-12 supplemented with 2 mM L-glutamine (Gibco), 10% fetal bovine serum (FBS), 1x GlutaMAX (Gibco), 100 units/ml penicillin, and 100 μg/ml streptomycin. CAD cells were differentiated in serum-free media prior to the experiments. The human SH-SY5Y cells (ATCC, CRL-2266), a DAergic cell line, were cultured in the same media as those for CAD cells and differentiated with 10 μM RA prior to the experiments (64). Cells were dissociated using 0.125% trypsin, 0.1 g/L EDTA (Gibco), then plated in 6-well, 24-well, and 96-well plates for multiple assays or 100 × 20 mm or 150 × 20 mm dishes for promoter activity, mRNA, or protein analysis. All cells were maintained at 37 °C in a 95% air, 5% CO incubator.

### Reactive oxygen species (ROS) assay (oxidative stress assay)

ROS were measured in accordance with the manufacturer's protocols. The generation of ROS, as an indicator of oxidative stress, was measured using a CM-H_2_DCFDA ROS molecular probe (Thermo Fisher Scientific). End-point product fluorescence was measured in each assay using the Spectramax i3x multimode microplate reader from Molecular Devices. Briefly, CAD neurons were washed with PBS and pre-treated with TX (1 μM, 6 h) prior to treatment of Mn (250 μM, 2 h) at 37 °C. Cells were washed, and 2.5 μM CM-H2DCFDA was added for 10 min. End-point fluorescence was determined at an excitation/emission wavelength of 485/527 nm.

### Cell viability assay

CAD or SH-SY5Y cells (2 × 10^4^/well) were grown in 96-well plates, then pre-treated with TX (1 μM) for 24 h, followed by Mn (250 μM) exposure for 24 h. Cells were also treated with a Wnt inhibitor LGK-974 (3 μM) for 12 h, followed by TX (1 μM) for 12 h, then exposed to Mn (250 μM) for 24 h. After incubation for the designated time period, cells were washed twice with ice-cold PBS. 100 μl of 3-(4,5-dimethylthiazol-2-yl)-2,5-diphenyltetrazolium bromide (MTT, 0.5 mg/ml in PBS) from MilliporeSigma was added, and cells were incubated for 3 h at 37 °C. After incubation, 100 μl of isopropanol was added, and the absorbance of the converted dye was measured at a wavelength of 570 nm using a microplate reader.

### Transient transfections

Cell transfections were carried out with Lipofectamine 3000 (Invitrogen, Thermo Fisher Scientific) in accordance with the manufacturer's guidelines. The transfection process to introduce plasmid vectors into cells was performed at a concentration of 0.5 μg per 5.0 × 10^5^ cells using Lipofectamine. After transfection, cells were left overnight before subsequent assays and analyses were performed.

### Measurement of promoter activity

CAD or SH-SY5Y cells were transfected with the human REST promoter plasmid using Lipofectamine 3000. After overnight transfections, the effects of various compounds on REST promoter activities were determined using the Bright-Glo luciferase assay kit (Promega, Madison, WI) according to the manufacturer's instructions.

### Western blot

After treatment with the designated compounds, CAD or SH-SY5Y cells were washed with ice-cold PBS. To extract the cellular proteins, cells were lysed with a radioimmunoprecipitation assay (RIPA) buffer containing a protease inhibitor (PI) cocktail, and the supernatant was collected after centrifugation at 13,000 rpm for 15 min at 4 °C. Equal amounts of proteins were loaded onto a 10% SDS-PAGE gel, followed by immunoblotting analysis with relevant antibodies. For visualization, all blots were treated with a West Pico PLUS chemiluminescence substrate detection kit (Pierce), followed by imaging and quantification of bands using the Bio-Rad ChemiDoc Imaging System and Image Laboratory Software version 5.2.1 (Bio-Rad) and ImageJ Software (Bethesda, MD) as described previously (16).

### Immunocytochemistry

CAD cells were grown on glass coverslips coated with poly-L-lysine in 6-well plates for immunostaining as described previously (16). β-catenin and REST were targeted using primary antibody with a dilution of 1:250 and a 1:1000 dilution of Alexa Fluor 488-conjugated secondary antibody. Leica SPEII confocal microscope (Leica Microsystems Inc.) was used to evaluate the cellular localization and fluorescence intensity of each sample.

### Preparation of cytoplasmic and nuclear fractions

The cells were lysed using fractionation buffer (20 mM HEPES, pH 7.4, 10 mM KCl, 2 mM MgCl_2_, 1 mM EDTA, 1 mM EGTA, and 1 mM DTT) containing PI cocktail, followed by mechanical lysis using a syringe and centrifugation at 720*g* for 5 min at 4 °C. The resulting lysates, which contained cytoplasmic fractions, were saved. The nuclei in the pellet were then resuspended in tris-buffered saline (TBS) with 0.1% sodium dodecyl sulfate (SDS). Protein concentrations in the resulting nuclear fractions were determined using a bicinchoninic acid (BCA) assay.

### Quantitative reverse transcription-polymerase chain reaction (qRT-PCR)

Following the appropriate treatment, samples from CAD cells were prepared for quantitative PCR (qPCR). To extract total RNA from cells, three samples per group were processed using the RNeasy Mini Kit (Qiagen). Purified RNA (2 μg) was then subjected to reverse transcription using the high-capacity cDNA reverse transcription kit (Applied Biosystems) to generate complementary DNA (cDNA). Real-time qPCR was performed on the CFX96 instrument (Bio-Rad) using iQ SYBR Green Supermix (Bio-Rad) and 0.4 μM forward and reverse primers. The following primers were utilized: REST (mouse) forward: 5′-ACT TTG TCC TTA CTC AAG CTC-3′, reverse: 5′-CAT TTA AAT GGC TTC TCA CCT G-3′; REST (human) forward: 5-′GTG AGC GAG TAT CAC TGG AGG-3′, reverse: 5-′CCC ATT GTG AAC CTG GTC TTG C-3′, β-catenin (mouse) forward: 5′-CTC GTG TCC TGT GAA GC-3′, reverse: TCA GCT TGA GTA GCC ATT G-3′, Dkk-1 (mouse) forward: 5′-TTG ACA ACT ACC AGC CCT AC-3′, reverse: 5′-AGG GCA TGC ATA TTC CAT TT-3′ Wnt3a (mouse), forward: 5′-AAC TGC ACC ACC GTC AGC AAC A -3′, reverse: 5′-AGC GTG TCA CTG CGA AAG CTA C-3′, ER-α (mouse) forward: 5′-TAC GAA GTG GGC ATG ATG AA-3′, reverse: 5′-AAG GTT GGC AGC TCT CAT GT-3′. GAPDH (mouse) forward: 5′-CTC ATG ACC ACA GTC CAT GC-3′, reverse: 5′-CAC ATT GGG GGT AGG AAC AC-3′. GAPDH (human) forward: 5-′TCC CTC AAG ATT GTC AGC AA-3′, reverse: 5′-AGA TCC ACA ACG GAT ACA TT-3′. The qPCR parameters consisted of 1 cycle at 95 °C for 10 min, followed by 40 cycles at 95 °C for 15 s and 60 to 65 °C for 1 min. The mRNA levels were analyzed using Bio-Rad CFX Manager Version 3.1, with GAPDH as the internal control.

### Chromatin immunoprecipitation (ChIP) assay

The ChIP assay was conducted using the EZ-ChIP kit from MilliporeSigma, following the manufacturer's instructions as described previously (16). Real-time qPCR was performed using primers targeting the WRE consensus site on the REST promoter (forward: 5′-CCA ACT TTT CCC CGC TCT-3′, reverse: 5′-GCG TCC TAC CCT CTG AAC TG-3′). The immunoprecipitated DNA was quantified by measuring percent (%) inputs from qPCR products using the Bio-Rad CFX Manager 3.1 software.

### Electrophoretic mobility shift assay (EMSA)

EMSA was conducted using a LightShift chemiluminescent kit from Thermo Fisher Scientific, following the manufacturer's instructions as described previously (16). The primer pairs utilized for targeting the WRE consensus site on the REST promoter were as follows: 5′-GAA AGT CCA GCA ACA AAG AAA AGG AGT TG-3′ and 5′-CAA CTC CTT TTC TTT GTT GCT GGA CTT TC-3′.

### Site-directed mutagenesis

The WRE binding sequence in the human 5′UTR REST promoter was mutated using a Q5 mutagenesis kit (New England Biolabs, Ipswich, MA) according to the manufacturer's instructions. The 5′UTR REST promoter (−3390/+1) subcloned into the pGL3 basic vector was used as the original template for mutation. The primer sets used for WRE mutant (WRE-mut) were 5′-GAA ACT CCA GC**G C**CA AAG AAA AGT AGT CGG AGA AGG AGC-3′ and 5′- GGG GGA GAG GTG GGG GAG-3′. The WRE mutant clones were confirmed by Primordium sequencing.

### Statistical analysis

All data were expressed as the mean ± standard deviation. The statistical analyses were performed using either Student's *t* test or one-way analysis of variance, followed by Tukey's *post hoc* tests using the GraphPad Prism software Version 9.0. A *p*-value of less than 0.05 (*p* < 0.05) was considered statistically significant.

## Data availability

All data that support this study are provided in the article.

## Supporting information

This article contains supporting information.

## Conflict of interest

The authors declare that they have no conflicts of interest with the contents of this article.
