# Supplementary material for: Tamoxifen induces protection against manganese toxicity by REST upregulation via the ER-α/Wnt/β-catenin pathway in neuronal cells
Source: J Biol Chem. 2025 Apr 23;301(6):108529. doi: 10.1016/j.jbc.2025.108529 (PMC12152632; doi:10.1016/j.jbc.2025.108529)
Supplement: Supplemental data [file mmc1.zip › Supplementary legends-031725.docx]

**Figure S1. TX treatment before (pre-) or after (post-) Mn exposure protects CAD cells from Mn toxicity.** CAD cells were treated with 1 µM TX either before or after exposure to 250 µM Mn for 24 hr. For pre-treatment, TX was applied 12 hr before Mn exposure. For the post-treatment regimen, TX was administered at various time points (10 min [0.2 hr], 30 min [0.5 hr], 1 hr, and 2 hr) after Mn exposure. CAD cell viability was assessed using the MTT assay. ***p<0.001, ^###^p<0.001 compared to control. ^@@@^p<0.001, ^@@^p<0.01, ^@^p<0.05 compared to each other. (One-way ANOVA followed by Tukey’s post hoc; n=3). The data shown are representative of 3 independent experiments.

**Figure S2. TX offered protection against Mn-induced toxicity in SH-SY5Y neuronal cells by upregulating REST through the activation of Wnt signaling. *A,*** SH-SY5Y neurons were transfected with a human 5’UTR-REST promoter vector and then exposed with 1 µM TX, followed by luciferase assay to measure REST promoter activity. ***B-C,*** SH-SY5Y neurons were treated with 1 µM TX, followed by measurement of REST mRNA using qPCR (B) and protein using western blot (C). ***D,*** SH-SY5Y neurons were treated with 1 µM TX for 3 hr prior to 250 µM Mn exposure for 6 hr (in the presence of TX), followed by measurement of total β-catenin protein. ***E,*** SH-SY5Y cells were treated with 3 µM LGK-974 for 12 hr, followed by 1 μM TX for 12 hr, then exposed to 250 µM Mn for 24 hr (in the presence of LGK-974 and TX). Subsequently, cell viability was assessed using an MTT assay. GAPDH and β-actin were used as loading controls of mRNA and protein, respectively. ***p<0.001, **p<0.01, *p<0.05, ^###^p<0.001, ^##^p<0.01, compared to control. ^@@@^p<0.001 compared to each other. (One-way ANOVA followed by Tukey’s post hoc, n=3). The data shown are representative of 3 independent experiments.
