# Supplementary figures and images for: Tamoxifen induces protection against manganese toxicity by REST upregulation via the ER-α/Wnt/β-catenin pathway in neuronal cells
Source: J Biol Chem. 2025 Apr 23;301(6):108529. doi: 10.1016/j.jbc.2025.108529 (PMC12152632; doi:10.1016/j.jbc.2025.108529)

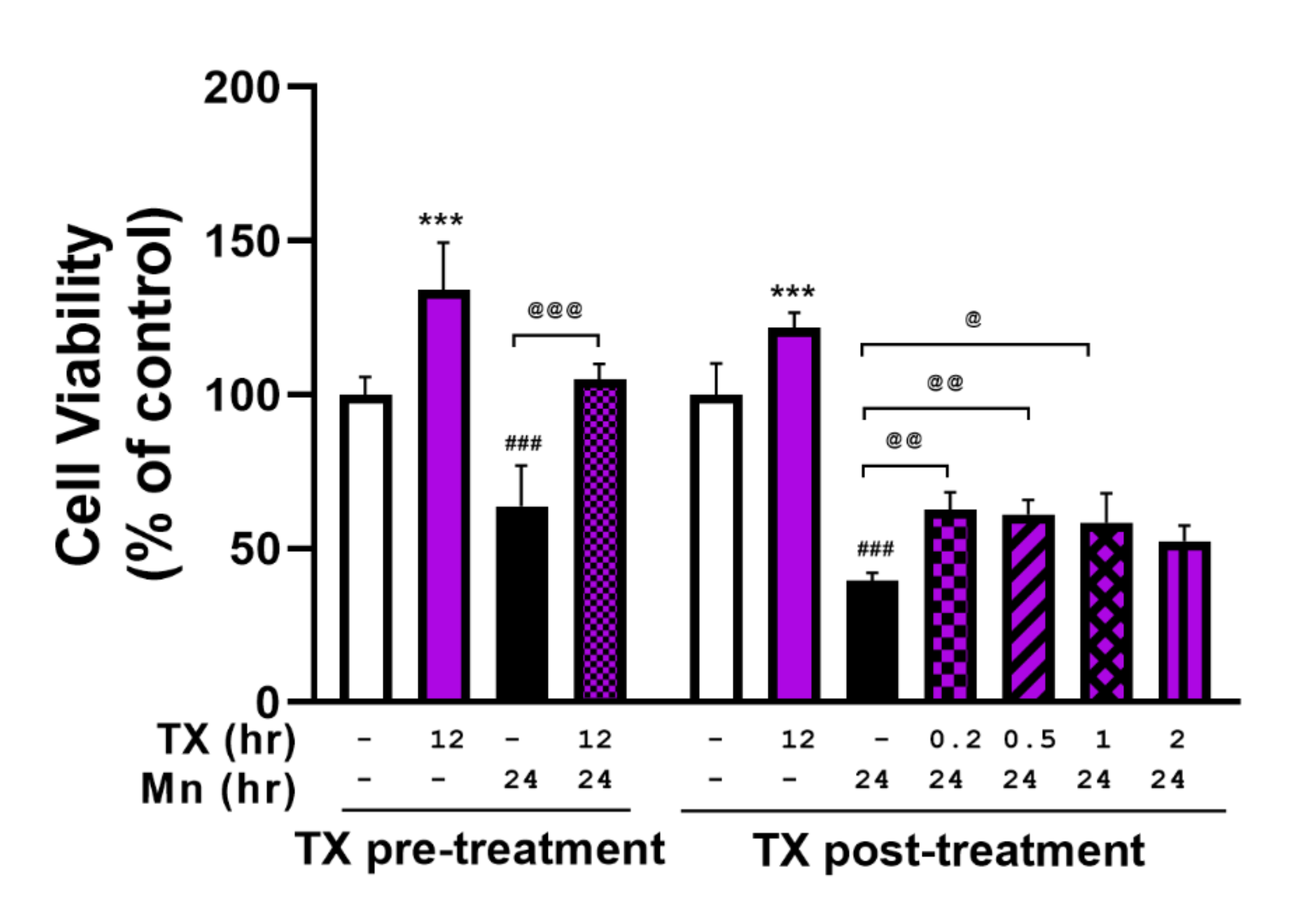

Supplement: Supplemental data [file mmc1.zip › Fig. S1.tif]

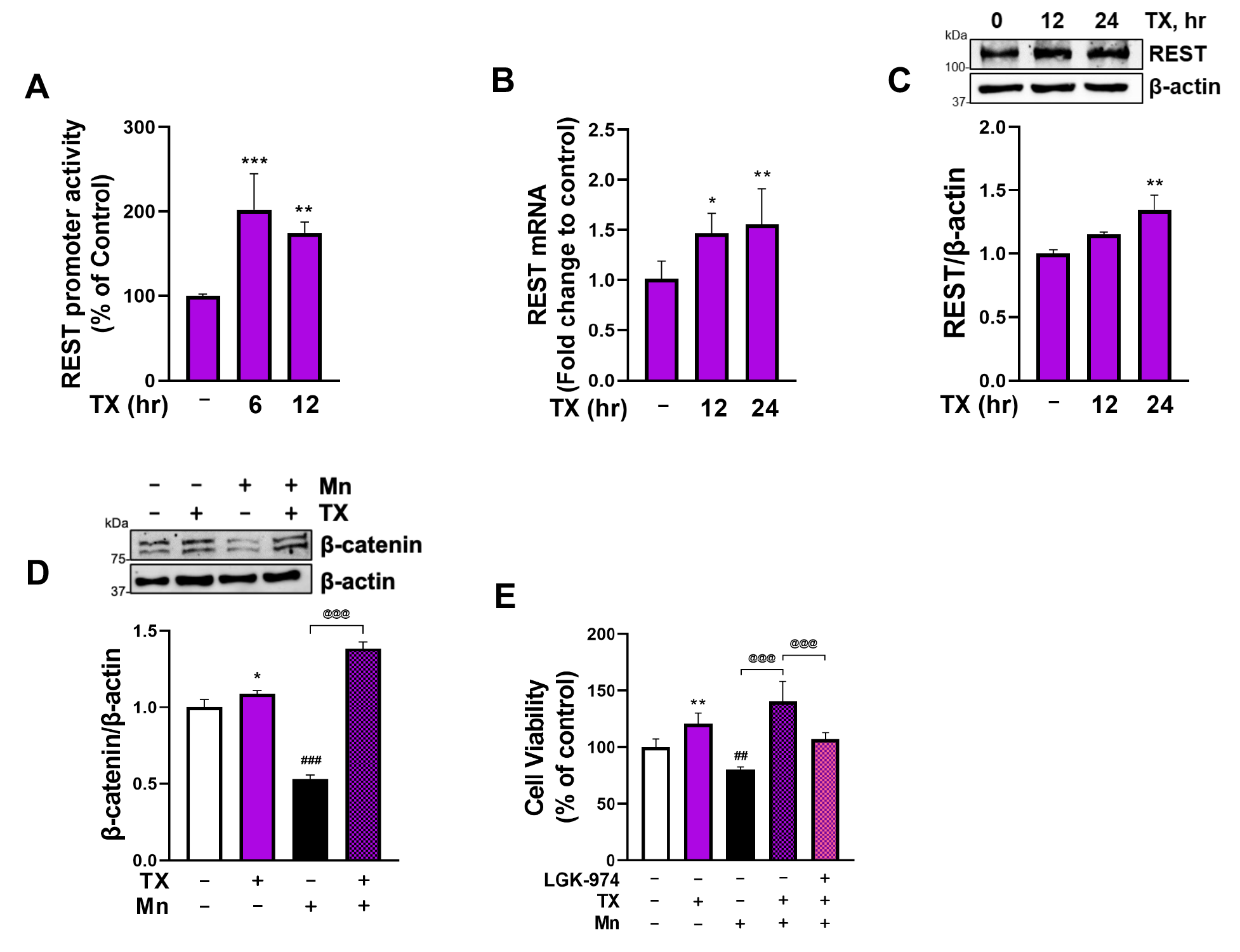

Supplement: Supplemental data [file mmc1.zip › Fig. S2.tif]
